# Laplacian spectra of a class of small-world networks and their applications

**DOI:** 10.1038/srep09024

**Published:** 2015-03-12

**Authors:** Hongxiao Liu, Maxim Dolgushev, Yi Qi, Zhongzhi Zhang

**Affiliations:** 1 School of Computer Science, Fudan University, Shanghai 200433, China; 2 Shanghai Key Laboratory of Intelligent Information Processing, Fudan University, Shanghai 200433, China; 3 Theoretical Polymer Physics, University of Freiburg, Hermann-Herder-Str.3, D-79104 Freiburg, Germany

## Abstract

One of the most crucial domains of interdisciplinary research is the relationship between the dynamics and structural characteristics. In this paper, we introduce a family of small-world networks, parameterized through a variable *d* controlling the scale of graph completeness or of network clustering. We study the Laplacian eigenvalues of these networks, which are determined through analytic recursive equations. This allows us to analyze the spectra in depth and to determine the corresponding spectral dimension. Based on these results, we consider the networks in the framework of generalized Gaussian structures, whose physical behavior is exemplified on the relaxation dynamics and on the fluorescence depolarization under quasiresonant energy transfer. Although the networks have the same number of nodes (beads) and edges (springs) as the dual Sierpinski gaskets, they display rather different dynamic behavior.

One of the most major problems in the study of networks is to understand the relations between their topology and the dynamics^1^. For instance, in the framework of generalized Gaussian structures (GGSs)^2^,^3^,^4^,^5^, the dynamics of polymer networks is fully described through the Laplacian eigenvectors and eigenvalues. In the field of GGSs and dynamical processes, the investigation of Laplacian eigenmodes has a paramount importance for the relaxation dynamics, the fluorescence depolarization by quasiresonant energy transfer^6^,^7^,^8^, the mean first-passage time problems^9^,^10^,^11^, and so on. Laplacian eigenvalues and eigenvectors play an irreplaceable role and they are also relevant to multi-aspects of complex network structures, like spanning trees^12^, resistance distance^13^ and community structure^14^. However, it is a challenging task to derive exact Laplacian eigenvalues or eigenvectors for a complex system and based on them to describe its dynamics. We remark that for this the use of deterministic structures is of much help^15^,^16^,^17^,^18^,^19^. Although the structural disorder leads in case of many real networks like hyperbranched polymers to smoothing-out and averaging, the topological features are still reflected in the typical scaling behaviors^20^. Furthermore, recently a striking development of chemistry made possible the synthesis of the hierarchical, fractal Sierpinski-type compounds^21^. Undoubtedly, this new achievement will keep the interest of the theorists on the regular structures, especially on those with loops.

The study of Laplacian eigenvalues has exhibited its activity during the past few decades, among extensive subjects and researches. The works from last century had solved the Laplacian eigenvalues for considerable amount of famous networks, like dual Sierpinski gaskets (in 2 or higher dimensions)^15^,^16^, dendrimers^17^, and Vicsek fractals^18^,^19^. Another type of model structures, which often arise in the complex systems or polymer networks, are the so-called small-world networks (SWNs)^22^,^23^,^24^,^25^. Recent studies have also suggested that SWNs play a notable role in real life^26^,^27^.

In this report we introduce a new kind of SWNs. Their construction is based on complete graphs consisting of *d* nodes and they have the same number of nodes and of edges as the dual Sierpinski gaskets embedded in (*d* − 1)-dimension. A complete graph is a simple undirected graph in which every pair of distinct vertices is connected by a unique edge. It has been widely used in quantum walks^28^,^29^, tensor networks^30^, social networks^31^, and explosive percolation problem^32^. While the SWNs introduced here are based on complete graph, their clustering coefficient shows that the SWNs are similar to complete graphs only in the limit *d* → ∞. As we proceed to show, also in this limit they have similar behavior as the dual Sierpinski gaskets embedded in to *d* → ∞ dimensions. On the other hand, for finite *d*, the SWNs display a macroscopically distinguishable behavior.

The report is organized as follows: First, we present the construction of SWNs, analyze their properties and their Laplacian spectra (the derivation of the recursive equations for the eigenvalues is given in Methods). Then, based on the spectra we consider the dynamics of networks, namely, the structural average of the mean monomer displacement under applied constant force and the mechanical relaxation moduli, and the dynamics on networks, exemplified through the fluorescence depolarization. Finally, we summarize and discuss our results.

## Results

### Model structures

**Figure 1:**
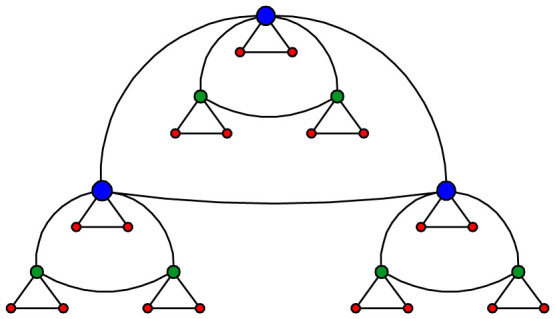
Construction of 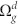 for *d* = 3 and *g* = 1 (blue beads), *g* = 2 (blue and green beads), *g* = 3 (all beads).

We start with a brief introduction to a family of small-world networks (SWNs) 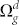 characterized by two parameters *d* and *g*, where *d* stands for the number of nodes of complete graph and *g* for the current generation. Figure 1 shows a construction process from 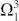 to 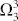: At first, 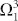 is a simple triangle, that is, a complete graph with 3 nodes. At the next stage, each node in 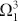 is replaced by a new complete graph. Thus each of the newly appeared complete graphs contains exactly one node of 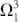 and we get the network at second generation 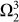. The growth process to the next generation continues in a similar way: Connecting a complete graph to each of the node of 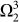 one gets 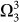. In general, we have *d^g^*^−1^ nodes at generation *g* − 1. By attaching *d* − 1 nodes to each existing node, increases their total number from *d^g^*^−1^ to *d^g^*. In this way, we get immediately the number of nodes in this network, 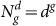, and the number of edges, 
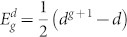
. It has to be mentioned that the dual Sierpinski gaskets embedded in (*d* − 1)-dimension have exactly the same number of nodes and of edges^33^.

To give evidence of the small-world property, we consider another characteristics, the diameter of the network. For a network, the diameter means the maximum of the shortest distances between all pairs of nodes in it^1^. Let 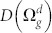 be the diameter of network 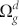. It is clearly that at generation *g* = 1, 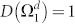. At each iteration *g* ≥ 1, new complete graphs are added to each vertex. Let us define the two nodes with longest distance in the existing network as *M_A_* and *M_B_*. It is easy to see that these two nodes belong to the complete graphs attached to *M_A_* and *M_B_*, respectively. Hence, at any iteration, the diameter of the network increases by 2 at most. Then the diameter of Ω*_g_* is just equal to 2*g* − 1, a result irrelevant to parameter *d*. The value can be presented by another form 2 log*_d_N_g_* − 1, which grows logarithmically with the network size indicating that the networks 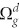 are small-world^1^.

**Figure 2:**
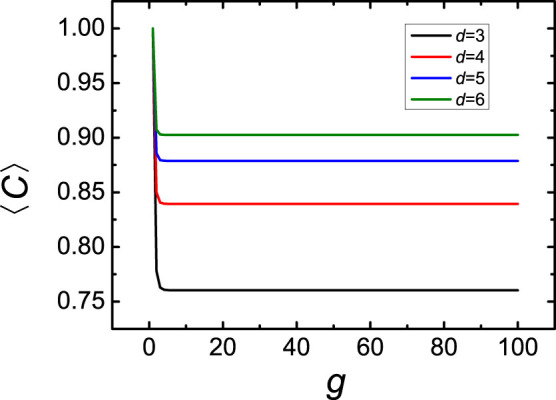
Clustering coefficients of 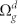 for the parameters *d* from 3 to 6, when *g* varies from 1 to 100.

Now we turn to the clustering coefficient of any node *i*, which is given by *C_i_* = 2*e_i_*/[*k_i_*(*k_i_* − 1)], where *e_i_* is the number of existing links between all the *k_i_* neighbors of node *i*^34^. From the network construction, we come to a simple conclusion that if node *x* exists for *h* generations, external (*d* − 1)*h* nodes will be attached to it. That is, *k_x_* = (*d* − 1)*h*. Among the (*d* − 1)*h* neighbors, *d* − 1 nodes that belong to the same complete graph are connected to each other, leading to the total number of links *e_x_* = *h*[(*d* − 1)(*d* − 2)/2]. Thus, the *C_x_* is given by
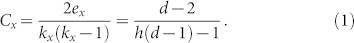
Based on equation (1) we can list the correspondence between each kind of clustering coefficient and the corresponding amount of nodes:
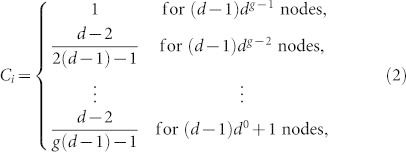
where the last situation represents the center of the whole network. Then we can obtain the average clustering coefficient of all the nodes,
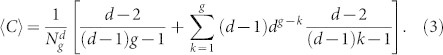


Figure 2 shows 〈*C*〉 as a function of *g* for *d* going from 3 to 6. As one can infer from the figure, 〈*C*〉 decreases very rapidly at small generations to a some constant value, which depends on *d*. In fact, one can find from equation (3) that for 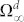 the average clustering coefficient is given by 〈*C*〉_∞_(*d*) = ((*d* − 1)/*d*)_2_*F*_1_[(*d* − 2)/(*d* − 1), 1; (2*d* − 3)/(*d* − 1); 1/*d*], where _2_*F*_1_[…] is the hypergeometric function, i.e. 〈*C*〉_∞_(3) ≈ 0.76, 〈*C*〉_∞_(4) ≈ 0.84, 〈*C*〉_∞_(5) ≈ 0.88, and 〈*C*〉_∞_(6) ≈ 0.9. For very large *d* (*d* → ∞), equation (3) converges to value 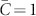, an inherent property of a complete graph.

### Recursion formulae for the Laplacian spectrum

Let 
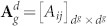
 denote the adjacency matrix of 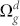, where *A_ij_* = *A_ji_* = 1 if nodes *i* and *j* are adjacent, *A_ij_* = *A_ji_* = 0 otherwise, then the degree of node *i* is 
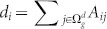
. Let 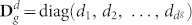 denote the diagonal degree matrix of 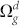, then the Laplacian matrix of 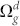 is defined by 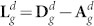.

To get a solution for the eigenvalues of 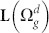, we have to concentrate our attention on its characteristic polynomial, 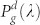. Here we just give a result and put off the proof and details in Methods:
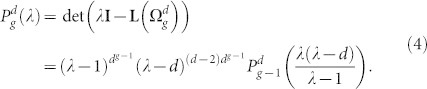
The recursion relation provided in equation (4) determines the eigenvalues of Laplacian matrix for 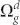. Note that 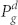 has a factor *λ* − *d* with exponent (*d* − 2)*d^g^*^−1^, i.e. equation (4) has the root *λ* = *d* with multiplicity at least (*d* − 2)*d^g^*^−1^.

It is evident that 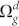 has *d^g^* Laplacian eigenvalues, denoted by 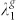, 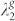, …, 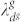, the set of which is represented by Λ*_g_*, i.e., 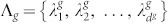. In addition, without loss of generality, we assume that 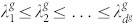. On the basis of above analysis, Λ*_g_* can be divided into two subsets 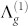 and 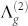 satisfying 
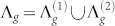
, where 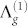 contains all eigenvalues equal to *d*, while 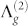 includes the remaining eigenvalues. Thus,
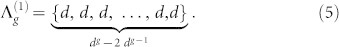


The remaining 2*d^g^*^−1^ eigenvalues belonging to 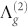 are determined by 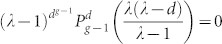. Let the 2*d^g^*^−1^ eigenvalues be 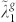, 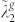, …, 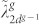, respectively. That is, 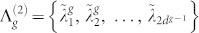. Given that the 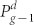 is the characteristic polynomial of 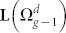 leading to *N_g_*_−1_ eigenvalues 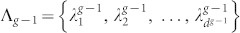, the set 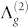 follows from
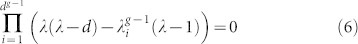
or from
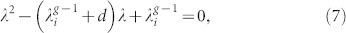
where *i* runs from 1 to *N_g_*_−1_ = *d^g^*^−1^.

Solving the quadratic equation (7), we obtain two roots 
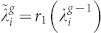
 and 
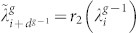
, where *r*_1_(*x*) and *r*_2_(*x*) are
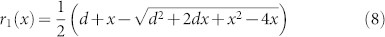
and
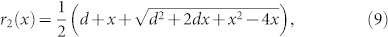
respectively. Thus, each eigenvalue 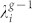 of Λ*_g_*_−1_ gives rise to two new eigenvalues in 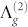 by inserting each Laplacian eigenvalue of Ω*_g_*_−1_ into equations (8) and (9). Considering the initial value 
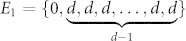
, by recursively applying equations (8) and (9) and accounting for 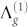, the Laplacian eigenvalues of Ω*_g_* are fully determined.

It is simple matter to check that equations (8) and (9) have the following behaviors:
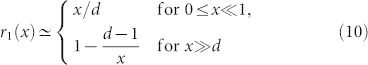
and
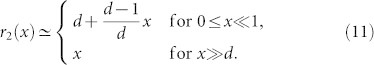
In this way equation (10) produces only small eigenvalues, *r*_1_(*x*) ∈ [0, 1) and equation (11) the large ones, *r*_2_(*x*) ∈ [*d*, ∞). Thus, the eigenvalue spectrum has always a gap [1, *d*), which is bigger for networks 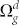 with larger *d*.

Now, it is interesting to examine the behavior of the small eigenvalues, i.e. to consider equation (10) for 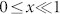. Our goal is to obtain the spectral dimension 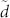 (also known as fracton dimension^35^). For this we use the methods of Ref. 36. Under equation (10) for 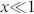, the *n* eigenvalues in the interval [*λ^g^*, *λ^g^* + Δ*λ^g^*] go over in *n* eigenvalues in the interval [*λ^g^*^+1^, *λ^g^*^+1^ + Δ*λ^g^*^+1^/*d*], while the total number of modes increases from *N* to *dN*. Hence, the density of states (modes) *ρ*(*λ*) for 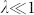 obeys
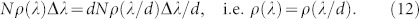
Using now the relation between *ρ*(*λ*) and the spectral dimension 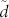
35,
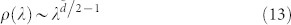
leads to
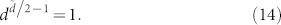
This means that the spectral dimension of the networks 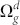 is 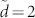 and 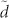 is independent on *d*. We note that for the dual Sierpinski gasket embedded in (*d* − 1)-dimension the spectral dimension is 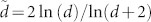, see e.g. Refs. 37, 38, i.e. it is similar to that of 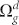 only in the limit *d* → ∞.

### Dynamics of polymer networks under external forces

We are going to study the networks 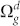 under the framework of generalized Gaussian structures (GGS)^3^,^4^,^5^, an extension of the classical Rouse beads-springs model^2^,^39^,^40^,^41^. Here we let all *N* beads of the GGS to be assigned to the same friction constant, *ζ*. The beads are connected to each other by elastic springs with spring constant *K*. The Langevin equation of motion for the *m*th bead in a system reads
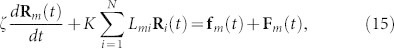
where **R***_m_*(*t*) = (*X_m_*(*t*), *Y_m_*(*t*), *Z_m_*(*t*)) is the position vector of the *m*th bead at time *t*, **L** describing the Laplacian matrix of the 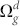. Moreover, **f***_m_*(*t*) is the thermal noise that is assumed to be Gaussian with zero mean value 〈**f***_m_*(*t*)〉 = 0 and 〈*f_mα_*(*t*)*f_nβ_*(*t*′)〉 = 2*k_B_Tδ_αβ_δ*_mn_δ(*t* − *t*′), where *k_B_* is the Boltzmann constant, *T* is the temperature, *α* and *β* represent the *x*, *y*, and *z* directions; **F***_m_*(*t*) is the external force acting on bead *m*.

First, we consider a quantity which is related to the micromanipulations with the polymer networks^42^. We put a constant external force **F***_k_*(*t*) = *F*Θ(*t*)*δ_mk_***e***_y_* (∀*k*), started to act at *t* = 0 (Θ(*t*) is the Heaviside step function) on a single bead *m* of the 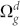 in the *y* direction. After averaging over all possibilities of choosing this monomer randomly, the displacement reads^4^,^5^,^39^
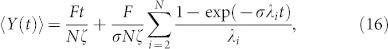
where *σ* = *K*/*ζ* is the bond rate constant, and *λ_i_* is the eigenvalues of matrix **L** with *λ*_1_ being the unique smallest eigenvalue 0.

Another example is the response to harmonically applied forces (strain fields), i.e. **F***_m_*(*t*) = *γ*_0_*e*^i*ωt*^*Y_m_*(*t*)**e***_x_*. The related response function is the so-called complex dynamic modulus G*(*ω*), or equivalently, its real *G*′(*ω*) and imaginary *G*″(*ω*) components (the storage and the loss moduli^41^,^43^). In the GGS model (for very dilute theta-solutions) the *G*′(*ω*) and *G*″(*ω*) are given by^3^
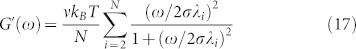
and
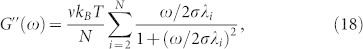
where *ν* denotes the number of polymer segments (beads) per unit volume.

**Figure 3:**
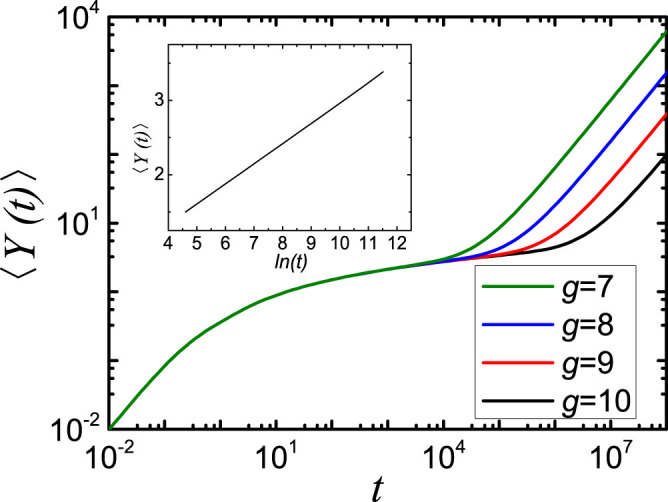
Averaged monomer displacement 〈*Y*(*t*)〉 for 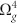, where *g* runs from 7 to 10.

We start by focusing on the averaged displacement 〈*Y*(*t*)〉, equation (16), where we set *σ* = 1 and 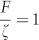. Figure 3 displays in double logarithmic scales the 〈*Y*(*t*)〉 for the networks 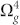 consisting of 4^7^ up to 4^10^ beads. As is known^4^,^5^,^39^, the 〈*Y*(*t*)〉 in such GGS at very long times reaches the domain 〈*Y*(*t*)〉 ~ *Ft*/(*Nζ*) and at very short times obeying 〈*Y*(*t*)〉 ~ *Ft*/*ζ*. However, in intermediate regime the network's beads move for several decades of time very slowly (logarithmic behavior^5^), up to the times *t* ~ *N* related to the diffusive motion of the whole structure. This differs from the corresponding patterns for the dual Sierpinski gaskets (embedded in (*d* − 1)-dimension)^37^,^38^, which show a slow subdiffusive behavior 〈*Y*(*t*)〉 ~ *t^α^* with *α* ≈ 0.23 for *d* = 4.

**Figure 4:**
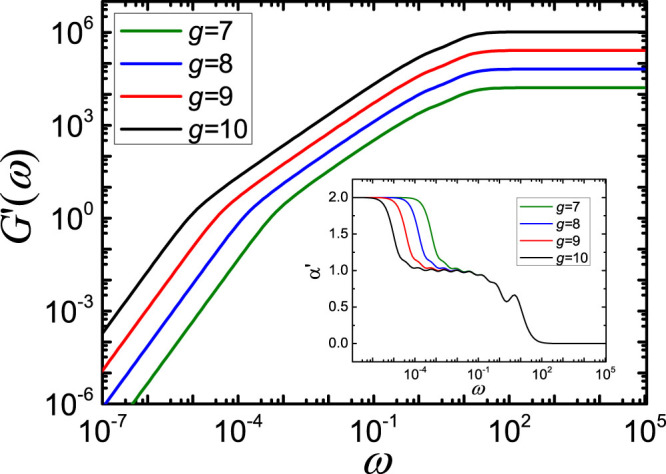
Storage modulus *G*′(*ω*) for 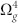, where *g* runs from 7 to 10.

While the 〈*Y*(*t*)〉 of 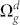 do not scale in the intermediate domain, the mechanical relaxation functions show in the related frequency domain a scaling behavior, see the results for storage moduli *G*′(*ω*) presented in Fig. 4. Here we plot them in dimensionless units by setting *σ* = 1 and 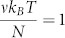. The networks are the same as for 〈*Y*(*t*)〉 of Fig. 3. The *G*′(*ω*) behaves commonly at very small and very high frequencies as *ω*^2^ and *ω*^0^, respectively. The in-between region of *G*′(*ω*) (related to the intermediate time domain of 〈*Y*(*t*)〉) the curves give in double-logarithmic scales the slopes around 1. This result is bigger than that in the same region of the corresponding dual Sierpinski gaskets embedded into 3-dimensional space (there one has slopes near 0.77)^37^. For a better visualization, we plot in the inset of Fig. 4 the effective slopes 
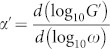
 for the same curves of Fig. 4. As expected, the limiting behaviors for very low and very high frequencies hold for slope 2 and slope 0. But in the intermediate frequency region, all of the four curves become wavy. Such a waviness reflects typically^36^,^37^,^38^ a very symmetric, hierarchical character of the structures. In case of real polymer systems, the inherit structural disorder smooths out such wavy patterns, while keeping the characteristic intermediate scaling^20^. Finally, the curves cross each other at the slope 1, keeping a short stable period and then falling into a value of 0.5.

### Fluorescence depolarization

We are now embarking on the dynamics of energy transfer over a system of chromophores^6^,^7^,^8^. As a usual way, we assume that the nodes (beads) only transfer their energy with their nearest neighbors. Under these conditions the dipolar quasiresonant energy transfer among the chromophores obeys the following equation^6^,^7^,^8^:
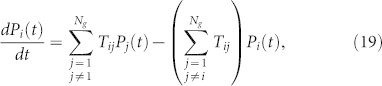
where *P_i_*(*t*) represents the probability that node *i* is excited at time *t* and *T_ij_* is the transfer rate from node *j* to node *i*. Following the framework of Refs. 6–8, we separate the radiative decay (equal for all chromophores) from the transfer problem, which can be included by the multiplication of all the *P_i_*(*t*) by exp(−*t*/*τ_R_*), where 1/*τ_R_* corresponding to the radiative decay rate. Under the assumption that all microscopic rates are equal to each other, fixed on a value 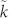, equation (19) becomes
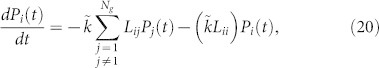
where *L_ij_* is the *ij*th entry of Laplacian matrix **L***_g_*. In equation (20) we used that for **L***_g_* the relation 
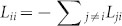
 holds.

The solution of equation (20) requires diagonalization of **L***_g_*. The result for a given *P_i_*(*t*) depends both on the eigenvalues and on the eigenvectors of **L***_g_*^6^,^7^,^8^. However, by averaging over all sites (a procedure fully justified when the dipolar orientations are independent of the beads' position in the system), the probability of finding the excitation at time *t* on the originally excited chromophore depends only on the eigenvalues of **L***_g_* and is given by^6^,^7^,^8^
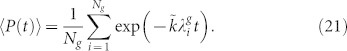


**Figure 5:**
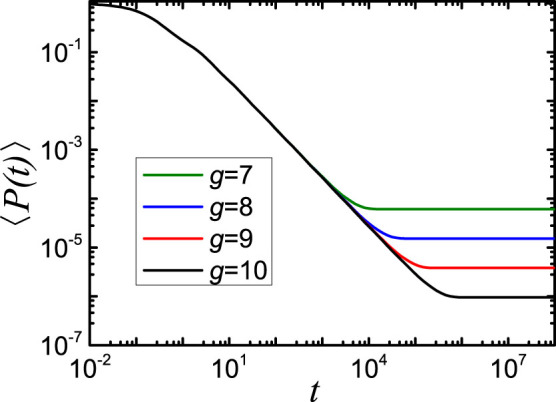
The average probability 〈*P*(*t*)〉, equation (21), for 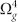, where *g* runs from 7 to 10.

Measuring the time in units of 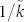, we can obtain the 〈*P*(*t*)〉 with 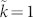. In Fig. 5 we display in double logarithmic scales the average probability 〈*P*(*t*)〉 that an initially excited chromophore of the network 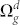 is still or again excited at time *t*. As for the previous figures, we choose *d* = 4 and change the generation *g* from 7 to 10, which means that the number of beads varies from 4^7^ to 4^10^. From Fig. 5 a waviness superimposed at early times can be observed immediately. Such waviness has been predicted in the regular hyperbranched fractals^6^ and it is related to high symmetry (regularity) of the network, i.e. the averaging due to possible disorder will smooth out the curves. Besides, in the intermediate time domain the decays show a power-law behavior, i.e. 〈*P*(*t*)〉 ~ *t*^−*α*^. In Fig. 5 the *α* float around 0.98 for all four generations, a very high value among similar kinds of networks.

**Figure 6:**
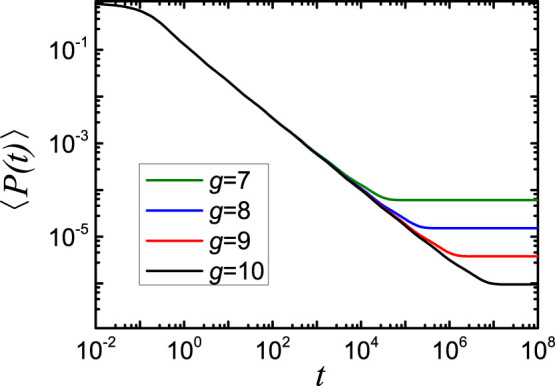
The average probability 〈*P*(*t*)〉, corresponding to the dual Sierpinski gaskets embedded into 3-dimension. The generation *g* runs from 7 to 10.

For the sake of comparison, in Fig. 6 we display the 〈*P*(*t*)〉 for dual Sierpinski gaskets embedded into 3-dimensional space for generations *g* as those in Fig. 5. What is clear from the figure, the curves also scale in the intermediate time domain, but have a smaller scaling exponent *α* = 0.78 compared to that of the networks introduced in this paper. Moreover, the four curves saturate to a constant value later than those of Fig. 5, while the plateau values 〈*P*(∞)〉 are exactly the same for both figures and equal to 1/*N_g_*^6^,^7^. This indicates that the equipartition of the energy over all beads is reached faster for the 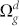 networks than for the dual Sierpinski gaskets with the same number of nodes and edges.

## Discussion

In summary, we have introduced a class of small-world networks constructed based on complete graphs. First, we have calculated the full Laplacian spectrum obtained from recursion formulae and proved its completeness. The corresponding analytic expressions allowed us to analyze the eigenvalues in detail and to calculate the related spectral dimension 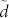. Using the eigenvalues, we have discussed the dynamics of such polymer networks in the GGSs framework, as well as the energy transfer through fluorescence depolarization. The ensuing spectral dimension 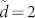 leaves its fingerprints in all quantities considered in the paper. In the intermediate time or frequency domain they follow the asymptotic relations^5^,^6^,^7^,^35^,^36^:
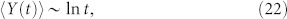

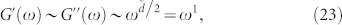

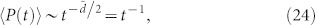
which were proven here by the numerical calculations. The networks introduced here are deterministic and highly structured, however, in case of a possible weak disorder leading to smoothing out of the curves the conclusions will still hold.

We believe that recent advances in the synthesis of fractal supramacromolecular polymers^21^ will open new perspectives for the compounds constructed based on the symmetric small-world networks presented in the report. Finally, we remark that we expect to find more applications of the networks considered here; in particular, the analytic expressions for the Laplacian eigenvalues determined here will be of much help.

## Methods

### Characteristic polynomial for the Laplacian eigenvalues of 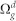


Following from the construction of 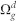, the adjacency matrix 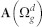, the degree matrix 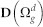, and the Laplacian matrix 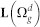 can be expressed as
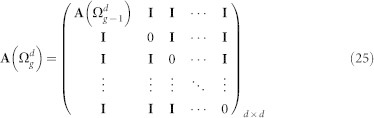

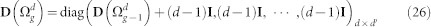
and
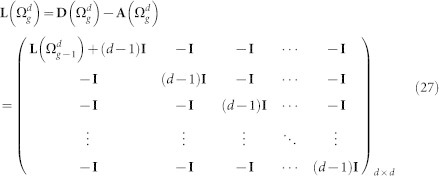


The characteristic polynomial of the 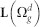 is determined as:
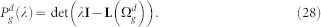
The matrix 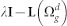 can be rewritten as:
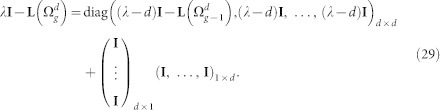


Now, using the matrix determinant lemma, see e.g. Ref. 44
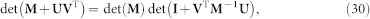
we obtain
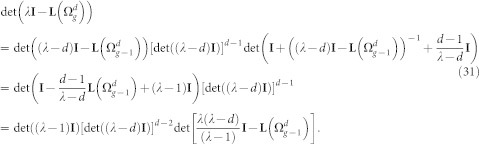
Thus,
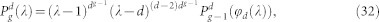
where
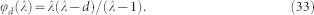


### Laplacian Eigenvectors of 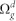


Analogous to the eigenvalues, the eigenvectors of 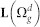 can also be derived directly from those of 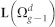. Assume that *λ* is an eigenvalue of Laplacian matrix for 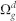, the corresponding eigenvector of which is *v* ∈ **R***^dg^*, where **R***^dg^* is the *d^g^*-dimensional vector space. Then the eigenvector *v* can be determined by solving equation 
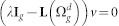
. We distinguish two cases: 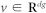 and 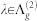, which will be separately treated as follows.

For the case of 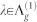, in which all *λ* = *d*, equation 
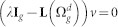
 becomes
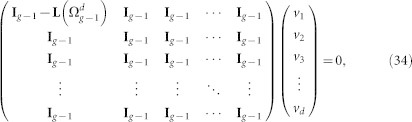
where vector *v_i_*(1 ≤ *i* ≤ *d*) are components of *v*. Equation (34) leads to the following equations:
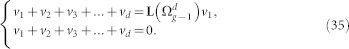
Then we know that *v*_1_ is the eigenvector corresponding to the eigenvalue 0 in 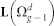, that is, 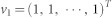. Let 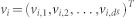, then, Eq. (35) is equivalent to the following equations:
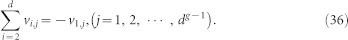
The set of all solutions to any of the above equations consists of vectors of the following form
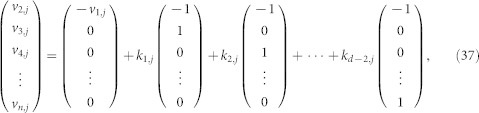
where *k*_1,*j*_, *k*_2,*j*_, …, *k_d_*_−2,*j*_ are arbitrary real numbers. In Eq. (37), the solutions for all the vectors *v_i_*(2 ≤ *i* ≤ *d*) can be rewritten as
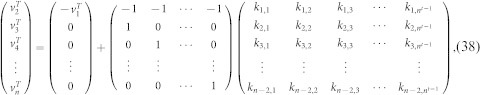
where *k_i_*_,*j*_(1 ≤ *i* ≤ *d* − 2; 1 ≤ *j* ≤ *d^g^*^−1^) are arbitrary real numbers. Using Eq. (38), we can obtain the eigenvector *v* associated with the eigenvalue *d*. Furthermore, we can easily check that the dimension of the eigenspace of matrix 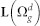 corresponding to eigenvalue *d* is (*d* − 2)*d^g^*^−1^.

We proceed to address the case of 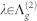. For this case, equation 
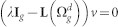
 can be rewritten as
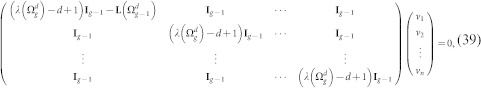
where vector *v_i_*(1 ≤ *i* ≤ *d*) are components of *v*. Eq (39) leads to the following equations:
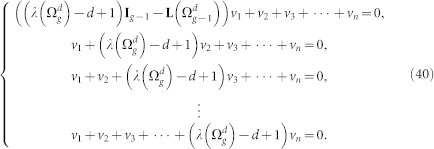
Resolving Eq. (40) yields
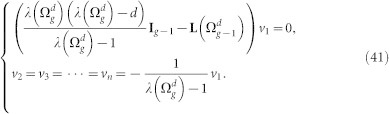


As demonstrated in the first subsection of Methods, if *λ* is an eigenvalue of 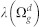, then 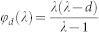 is an eigenvalue of 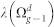. When *i* ≤ *d^g^*^−1^, we have 
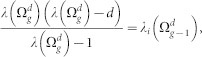
, while in the situation *d^g^*^−1^ < *i* ≤ 2*d^g^*^−1^, 
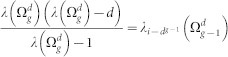
. From Eq. (41), vector *v*_1_ is the eigenvector of 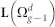 corresponding to the eigenvalue 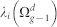. Applying the *v*_1_ into Eq. (41), we will get all of the *v_i_*(2 ≤ *i* ≤ *d*) and finally the eigenvector of 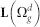 corresponding to 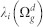. In this way, we have completely determined all eigenvalues and their corresponding eigenvectors of 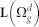.

## Author Contributions

H.L., M.D. and Z.Z.Z. designed the research. H.L. and Y.Q. performed the research. H.L., M.D. and Z.Z.Z. wrote the manuscript.
